# Complete Genome Sequences of Two Novel Isolates of Human Parainfluenza Virus 1 Associated with Acute Respiratory Infection

**DOI:** 10.1128/genomeA.01154-16

**Published:** 2016-10-13

**Authors:** J. L. Denson, J. L. Kennedy, W. N. Dehority, M. M. Eickman, K. S. Schwalm, A. N. Stoner, J. C. Kincaid, T. J. Abramo, T. M. Thompson, E. M. Ulloa, S. W. Burchiel, S. A. Young, D. L. Dinwiddie

**Affiliations:** a Department of Pharmaceutical Sciences, College of Pharmacy, University of New Mexico Health Sciences Center, Albuquerque, New Mexico, USA; b Department of Pediatrics, University of New Mexico Health Sciences Center, Albuquerque, New Mexico, USA; c Department of Pediatrics, Arkansas Children’s Research Institute, University of Arkansas for Medical Sciences, Little Rock, Arkansas, USA; d TriCore Reference Laboratories, Albuquerque, New Mexico, USA; e Clinical Translational Sciences Center, University of New Mexico Health Sciences Center, Albuquerque, New Mexico, USA; f Clinical and Translational Sciences Center, University of Arkansas for Medical Sciences, Little Rock, Arkansas, USA

## Abstract

Using target capture of viral nucleic acid and next-generation sequencing, we generated the complete genomes of two novel human parainfluenza virus 1 isolates. Isolates AR001 (accession no. KX570602) and NM001 (accession no. KX639498) were collected 3 months apart from pediatric patients with acute respiratory infection from Arkansas and New Mexico, respectively.

## GENOME ANNOUNCEMENT

Acute respiratory infections (ARI) are one of the leading causes of morbidity and mortality in young children, accounting for ~25% of hospital admissions (1) and almost 2 million deaths worldwide per year (2, 3). Human parainfluenza viruses (hPIV) are one of the leading causes of ARI and pediatric hospitalizations in the United States (4, 5). The hPIVs are enveloped viruses with a single-stranded negative-sense RNA genome of ~15,300 to 17,400 nucleotides that belong to the subfamily *Paramyxovirinae* of the *Paramyxoviridae* family (6). There are four distinct serotypes of hPIVs (hPIV-1, -2, -3, and -4a/b) (7).

Here, we present the complete genome sequences of two novel hPIV1 isolates collected 3 months apart from two pediatric patients with differing clinical courses. Patient 1 (AR001) was a 5-year-old male with no significant past medical history seen at Arkansas Children’s Hospital in Little Rock, AR, on 9 September 2015 after having a nonproductive cough for 6 days with rhinorrhea. The patient was afebrile and had an oxygen saturation in arterial blood (SaO_2_) of 98%. A chest X ray revealed cardiopulmonary disease. He required only supportive care and was discharged home after evaluation. Patient 2 (NM001) was a 6-year-old otherwise-healthy female who presented on 16 December 2015 to the University of New Mexico Hospital in Albuquerque, NM, with 10 days of productive cough, intermittent fevers up to 104°F, and a SaO_2_ of 89%. Her chest X ray documented bronchial wall thickening and hyperinflation consistent with a viral infection. She was hospitalized for one day on the general pediatric floor and required 5 h of supplemental oxygen.

An Illumina stranded-RNA sequencing library was created from RNA isolated from swab samples, and hybridization-based enrichment was performed using the University of New Mexico (UNM) ResVir respiratory viral panel probe set. The UNM ResVir panel contains 5,683 hybridization probes designed to be complimentary to coding sequence (CDS) regions of 24 human respiratory viruses. Next-generation sequencing was performed on an Illumina MiSeq using v3 chemistry and paired 75-bp reads. Sequencing of AR001 and NM001 resulted in 83,471 and 919,050 sequencing reads aligning to the hPIV 1 RefSeq reference NC_003461, respectively. No other viruses were detected. Consensus genome sequences were generated by reference-guided alignment to NC_003461 and annotated using the VIPR Genome Annotator (8).

The genome of AR001 is 15,572 nucleotides in length and differs from the hPIV1 RefSeq reference NC_003461 by 647 nucleotides and 137 amino acids. Isolate NM001 is 15,580 nucleotides in length and differs from the hPIV1 RefSeq reference NC_003461 by 688 nucleotides and 133 amino acids. The two isolates differ from each other by 118 nucleotides and 23 amino acids. Phylogenetic analysis of the two isolates through nearest-neighbor joining (CLC Genomics Workbench version 9) revealed that both AR001 and NM001 grouped with two strains isolated in France in 2010 to 2011 (accession numbers KF530218 and KF530224) and a 2011 strain from the United States (accession no. KF530213). Taken together, these data indicate that although related, these two hPIV1 isolates, obtained from Arkansas and New Mexico 3 months apart, exhibit significant genomic differences, which may underlie the patients’ different clinical phenotypes.

### Accession number(s).

The whole-genome sequence of isolates AR001 and NM001 have been deposited in GenBank under the accession numbers KX570602 and KX639498, respectively.

## References

[1] Hasegawa K, Tsugawa Y, Brown DF, Mansbach JM, Camargo CA 2013 Trends in bronchiolitis hospitalizations in the United States, 2000–2009. Pediatrics 132:28–36. doi:10.1542/peds.2012-3877. 23733801 PMC3691534

[2] Bellos A, Mulholland K, O’Brien KL, Qazi SA, Gayer M, Checchi F 2010 The burden of acute respiratory infections in crisis-affected populations: a systematic review. Confl Health 4:3. doi:10.1186/1752-1505-4-3. 20181220 PMC2829474

[3] Nair H, Simões EA, Rudan I, Gessner BD, Azziz-Baumgartner E, Zhang JS, Feikin DR, Mackenzie GA, Moïsi JC, Roca A, Baggett HC, Zaman SM, Singleton RJ, Lucero MG, Chandran A, Gentile A, Cohen C, Krishnan A, Bhutta ZA, Arguedas A 2013 Global and regional burden of hospital admissions for severe acute lower respiratory infections in young children in 2010: a systematic analysis. Lancet 381:1380–1390. doi:10.1016/S0140-6736(12)61901-1. 23369797 PMC3986472

[4] Berman S 1991 Epidemiology of acute respiratory infections in children of developing countries. Rev Infect Dis 13(Suppl 6):S454–S462. doi:10.1093/clinids/13.Supplement_6.S454. 1862276

[5] Fowlkes A, Giorgi A, Erdman D, Temte J, Goodin K, Di Lonardo S, Sun Y, Martin K, Feist M, Linz R, Boulton R, Bancroft E, McHugh L, Lojo J, Filbert K, Finelli L, IISP Working Group 2014 Viruses associated with acute respiratory infections and influenza-like illness among outpatients from the Influenza Incidence Surveillance Project, 2010–2011. J Infect Dis 209:1715–1725. doi:10.1093/infdis/jit806. 24338352 PMC5749912

[6] Wang L, Collins P, Fouchier R, Kurath G, Lamb R, Randall R, Rima B 2012 Family *Paramyxoviridae*, p 672–685. *In* King AMQ, Lefkowitz E, Adams MJ, Carstens B (ed), Virus taxonomy: ninth report of the International Committee on Taxonomy of Viruses. Elsevier, Waltham, MA.

[7] Henrickson KJ 2003 Parainfluenza viruses. Clin Microbiol Rev 16:242–264. doi:10.1128/CMR.16.2.242-264.2003. 12692097 PMC153148

[8] Pickett BE, Sadat EL, Zhang Y, Noronha JM, Squires RB, Hunt V, Liu M, Kumar S, Zaremba S, Gu Z, Zhou L, Larson CN, Dietrich J, Klem EB, Scheuermann RH 2012 ViPR: an open bioinformatics database and analysis resource for virology research. Nucleic Acids Res 40:D593–D598. doi:10.1093/nar/gkr859. 22006842 PMC3245011

